# Test of the FlashFREEZE unit in tissue samples freezing for biobanking purposes

**DOI:** 10.1007/s10561-022-10045-1

**Published:** 2022-10-30

**Authors:** Edyta Biskup, Lone Schejbel, Douglas Nogueira Perez de Oliveira, Estrid Høgdall

**Affiliations:** Department of Pathology, Copenhagen University Hospital, Herlev, 2730 Denmark

**Keywords:** FlashFREEZE, Biobanking, Tissue freezing procedures, Fresh frozen tissue samples

## Abstract

Availability of molecularly intact biospecimens is essential in genetic diagnostics to obtain credible results. Integrity of nucleic acids (particularly RNA) may be compromised at various steps of tissue handling, and affected by factors such as time to freeze, freezing technique and storing temperature. At the same time, freezing and storing of the biological material should be feasible and safe for the operator. Here, we compared quality of DNA and RNA from biospecimens derived from different organs (breast, colon, adrenal glands, testes, rectum and uterus) frozen either using dry ice-cooled isopentane or with FlashFREEZE unit, in order to verify if the latter is suitable for routine use in biobanking. Implementing FlashFREEZE device would enable us to limit the use of isopentane, which is potentially toxic and environmentally harmful, whilst facilitate standardization of sample freezing time. We considered factors such RNA and DNA yield and purity. Furthermore, RNA integrity and RNA/DNA performance in routine analyses, such as qPCR, next generation sequencing or microarray, were also assessed. Our results indicate that freezing of tissue samples either with FlashFREEZE unit or isopentane ensures biological material with comparable expression profiles and DNA mutation status, indicating that RNA and DNA of similar quality can be extracted from both. Therefore, our findings support the use of the FlashFREEZE device in routine use for biobanking purposes.

## Introduction

Rapid collection and efficient processing of tissue samples are essential for molecular testing, such as mutation profiling or RNA expression, which facilitates therapeutic decisions in oncology. Fresh frozen samples used for molecular testing usually lack the information about tissue architecture (including tumor burden), and therefore are currently used to a lesser extent in routine diagnostics than formalin-fixed paraffin-embedded (FFPE) blocks. However, they preserve nucleic acids significantly better than FFPE (Shabihkhani et al. 2014; Chen et al. 2014; Newton et al. 2020) and hence are already widely used in research. Moreover, as molecular diagnostics utilizes increasingly complex tools and provides increasing amounts of information fresh frozen tissue may in the future present a real alternative to FFPE in molecular testing. However, the right freezing technique is crucial to ensure data quality.

In our facility, patient-derived samples are handled according to standard operating procedures of Danish Cancer Biobank, which include several preservation protocols depending on the downstream analysis, i.e. embedding in OCT cryogel, fixation in formalin followed by paraffine embedding (both for histological examination), RNAlater specifically for RNA extraction, or snap-freezing for DNA/RNA molecular testing. The latter is routinely performed in our diagnostic setting using cold isopentane (2-methylbutane). This method is robust and feasible, though not without shortcomings. Importantly, isopentane requires special handling, being extremely flammable both as a liquid and vapor, with a flash point as low as -51 °C. Although not mutagenic, it may be fatal when swallowed and cause drowsiness or dizziness when inhaled. It is toxic to aquatic organisms, and special precautions should be taken to avoid it being released into the environment (URL:https.//pubchem.ncbi.nlm.nih.gov/compound/Isopentane). Moreover, the procedure is difficult to standardize,. as the time spent in the isopentane container differs between samples. Samples prepared earlier are kept in the container until the entire batch is processed, irrespective of their weight. Finally, since the system is not closed, liquid loss due to evaporation will gradually occur, which may lead to temperature variance.

FlashFREEZE is a benchtop device, designed to freeze tissue samples for biobanking. It allows the use of various formats such as single tubes and 24-, 48- or 96-well plates and is claimed to be suitable for different kinds of biospecimens. Importantly, FlashFREEZE does not require either liquid nitrogen or isopentane. Instead it uses Novec™ 7000 (1-methoxyheptafluoropropane), a cooling liquid which is not combustible, nonconductive, and characterized by low toxicity and low Global Warming Potential (GWP; 3 M™ manufacturer declaration). Alternatively, 99% denatured ethanol can be used as a coolant.

The aim of this study was to verify whether implementing FlashFREEZE in our routine clinical biobanking, instead of the current snap-freezing by isopentane, may be warranted. To our knowledge, there is no study which would compare characteristics of tissue preserved with these two freezing methods. From here on, the “FlashFREEZE” term will refer to the tabletop freezing system FlashFREEZE plus the Novec7000 freezing medium.

## Materials and methods

### Human tissue samples

Material used in this study consists of fresh-frozen tissue cancer specimens, obtained for diagnostic purposes by the Department of Pathology, Herlev and Gentofte Hospital, Denmark, and stored at the Danish Cancer Biobank (DCB; Bio- and Genome Bank Denmark; RBGB; www.rbgb.dk). Tissue samples from the following organs were included: breast, colon, testis, rectum, uterus and adrenal glands. The overview of all the samples used in the study, including the tissue of origin and the end analysis, is gathered in Table 1. All tissue samples were collected and handled according to standard operating procedures, implemented and routinely performed by the Danish Cancer Biobank. Briefly, following excision, each sample intended for storage was split into smaller fractions of similar size, four of which were subjected to dry snap-freezing either in cold isopentane or with the FlashFREEZE unit (Milestone Medical, Sorisole, Italy). Time between tissue collection and freezing ranged from 27 min to 16.5 h, with a median of 1.25 h. Samples were typically frozen within 2 h after excision. In few cases (3 pairs of colon cancer tissue), due to logistic reasons (transportation from a local hospital), samples were kept overnight at 4 °C and frozen only the next day. These three samples were only used for yield comparison, not for downstream analyses, and have therefore no influence on the results from downstream analyses.

**Table 1:** Overview of the cancer tissue samples used in the study and of the analyses performed. Numbers refer to pairs of tissue samples (isopentane–FlashFREEZE)

	Total	Breast	Colon	Testis	Rectum	Corp. uteri	Adr. glands
Part I*: (only samples extracted with Protocol_O**)	34	22	12				
Preliminary comparison of the DNA/RNA yield	34	22	12				
Part II*: (only samples extracted with Protocol_P***)	27	12	8	3	2	1	1
Comparison of yield and purity (only samples where both RNA and DNA were available were included)	23	12	6	2	2	1	
Analysis of RNA integrity (in brackets, samples which gave a conclusive (28S/18S ratio)	23 (15)	12 (7)	6 (3)	2 (2)	2 (2)	1 (1)	
Oncomine Focus Assay (DNA)	5		5				
Oncomine Focus Assay (RNA)	5		5				
Samples used for qPCR BRAF mutation analysis (DNA)	10	4	3	1	1		1
Samples used for qPCR PIK3CA mutations analysis (DNA)	1	1					
Affymetrix GeneChip Array (RNA)	1	1					
Ion Ampliseq transcriptome analysis (NGS)	1	1					
Microsatellite instability analysis	2			1		1	

*Material used in Part I and Part II partly overlaps

**AllPrep DNA/RNA #80,204 (*Protocol_O*)

***AllPrep DNA/RNA/miRNA Universal kit #80,224 (*Protocol_P*; both from QIAGEN; Hilden, DE; see section: Nucleic acid extraction

Sample pairs intended for comparison were processed simultaneously.

Noteworthy, there was no access to the patient information prior to analyses, which meant that the original diagnostic results were unknown to us and sample pairs could not be selected for analyses based on known positive results.

### Freezing in dry ice-cooled isopentane

Prior to freezing, blocks of dry ice were portioned and positioned into the cooling tank, around the inner container. After equilibrating the temperature, the container was filled with ca. 200–300 mL isopentane (VWR Chemicals, Solon, OH). Then, each excised tissue sample was inserted into a cryovial and immediately placed in isopentane container. Of note, tissue fractions were added continuously to the isopentane and therefore time in isopentane may vary even within the individual patient sample.

### Freezing using FlashFREEZE unit

The preparation phase comprised of cooling the instrument down for approximately 2 h, filling the liquid container with up to 400 mL coolant Novec 7000 and allowing it to equilibrate for additional 15 min. Then, each excised tissue sample was put inside a cryovial and placed into the tank containing the coolant agent for 2 min. Samples were prepared individually. Thereafter, samples were placed into the cryovial holder compartment, and transferred to − 80 °C freezer at the end of the day. Importantly, unlike isopentane, Novec 7000 can also be used on the following days, providing sufficient volume. Hence, consumption of the coolant is lower than for isopentane freezing protocol.

### Nucleic acid extraction

Total RNA and DNA were extracted from the tissue samples using either AllPrep DNA/RNA #80204 (*Protocol_O*) or AllPrep DNA/RNA/miRNA Universal kit #80224 (*Protocol_P*; both from QIAGEN; Hilden, DE). Briefly, small fragments of fresh frozen specimens (not exceeding 3 mm^3^) were immersed in 150 µL RLT Plus buffer, containing 0.1% v/v *β*-mercaptoethanol, and disrupted using a pestle (Thermo Fisher Scientific, Waltham, MA). After initial disruption, 450 µL RLT Plus Buffer was added and samples were lysed for additional 5 min, before continuing with one of the two commercial kits, according to manufacturer’s instructions. When processed, tissue specimens were kept on dry ice prior to addition of RLT lysis buffer. Elution volume was 24 µL water or 100 µL EB buffer for RNA and DNA, respectively.

Nucleic acid concentration was quantified by Qubit® 2.0 Fluorometer with Qubit™ dsDNA HS Assay Kit or Qubit™ RNA HS Assay Kit, accordingly. Sample purity was assessed by 260 nm/280 nm absorbance ratio using NanoDrop™ One (all from Thermo Fisher Scientific).

### Analysis of RNA integrity

RNA integrity was evaluated by microcapillary electrophoresis using Agilent 2100 Bioanalyzer with Agilent RNA 6000 Nano Kit (Agilent technologies, Santa Clara, CA), according to manufacturer’s instructions. RNA Integrity was expressed as 28S/18S peak ratio.

### Quantitative PCR (qPCR)

DNA qPCR was performed using BRAF and PIK3CA mutations detection kits (EntroGen BRAF Mutation Analysis Kit II, BRAFX-RT64 and EntroGen PIK3CA mutation analysis kit for Real-Time PCR, PI3K-RT48, EntroGen, Woodland Hills, CA) and analyzed on the ABI 7500 Fast Real Time PCR system (Thermo Fisher Scientific).

Linearity and efficiency of the PCR amplification reactions were evaluated on the internal control gene amplification in the *BRAF* mutation detection kit as follows. Samples were subjected to tenfold DNA serial dilutions (20, 2 and 0.2 ng DNA per sample) followed by qPCR reaction, according to manufacturer’s protocol (EntroGen). The lm (linear model) function (package stats, R version 3.6.1) was used to find the linear relation between the DNA input (log_10_ of DNA concentration) and Ct values. Two parameters, (a) coefficient of determination (*R*^2^), measuring the goodness-of-fit in a linear regression, and (b) the slope were extracted. The slope was subsequently used to calculate the PCR reaction efficiency (E), using the formula: E = −1 + 10^(−1/slope)^.

For *PIK3CA* mutation detection, 10 ng DNA was used for each qPCR reaction, carried according to manufacturer’s protocol (EntroGen).

### Affymetrix Gene Chip Array

Samples were analyzed with the GeneChip™ Human Genome U133 Plus 2.0 assay, following manufacturer’s instructions (ThermoFisher Scientific). Data analysis was done with the Transcriptome analysis Console v4.0.2.15, Summarization Method: Robust Multi-array Average (RMA), Condition (Comparison): FlashFREEZE; Isopentane.

#### NGS

The Ion Torrent Oncomine Focus Assays for both DNA and RNA were performed following the manufacturer’s instructions (Thermo Fisher Scientific) using manual library preparation, the Ion Chef System for template preparation and chip loading, and sequencing on the Ion S5XL system with either 400 or 500 flows. Data analysis was performed in the Ion Reporter software with Oncomine Focus—w2.6 – DNA-Single Sample corrected End repair r. 0 and Oncomine Focus—w2.7—Fusions-Single Sample r. 0 workflows and default data filtering. The NGS Focus DNA panel results were evaluated on Read length histogram, Median Absolute Pairwise Difference (MAPD) and called variants. NGS Focus fusion panel results were evaluated on the expression of five internal expression controls, on the analysis software QC evaluation [PASS: Total Mapped Fusion Panel Reads > 5000; MeanReadLength > 0] and on fusion overall call.

The Ion AmpliSeq Transcriptome Human Gene Expression analysis was performed as instructed by the manufacturer (Thermo Fisher Scientific) using manual library preparation, the Ion Chef System for template preparation and chip loading and sequencing on the Ion S5XL system with 500 flows. Data were transformed to reads/million with the ampliSeqRNA—v5.16.0.0 plugin in the Torrent Server S5XL software and CHP files were imported to the Transcriptome analysis Console v4.0.2.15 for comparative analysis, Condition (Comparison): FlashFREEZE Isopentane.

### Microsatellite instability analysis

Microsatellite instability (MSI) analysis was done with the Pentabase PlentiPlex™ MSI Classic panel as instructed by the manufacturer (PentaBase) and analyzed on the ABI 3130XL Genetic analyzer (Thermo Fisher Scientific). It is a multiplexed MSI assay for the length analysis of five mononucleotide microsatellite loci (BAT-25, BAT-26, NR-21, NR-22 and NR-24).

### Statistical analysis

Comparison between isopentane and FlashFREEZE sample groups was performed either using two-sided* t*-test or Wilcoxon signed rank test (for non-gaussian distributions), with a significance level alpha = 0.05. Normality of data distribution was verified by quantile–quantile plot. Comparison of RNA and DNA amounts extracted from tissue samples derived from different organs was performed using Kruskal–Wallis one-way analysis of variance. Statistical analysis and graph preparation were made in RStudio using R programing language (version 3.6.1).

## Results

In order to test the use of FlashFREEZE unit for biobanking purposes we analyzed altogether 104 tissue samples from biopsies, i.e. 52 isopentane–FlashFREEZE tissue sample pairs (see Table 1), taking under consideration the nucleic acids (total RNA and DNA) yield, purity and integrity, and their performance in downstream analyses. Information of time-to-freeze was retrieved from DCB (see Materials and methods), and since isopentane and FlashFREEZE samples were prepared in parallel, their time-to-freeze was identical.

### RNA and DNA yield extracted from biospecimens frozen using isopentane or FlashFREEZE unit is comparable

**Fig. 1:**
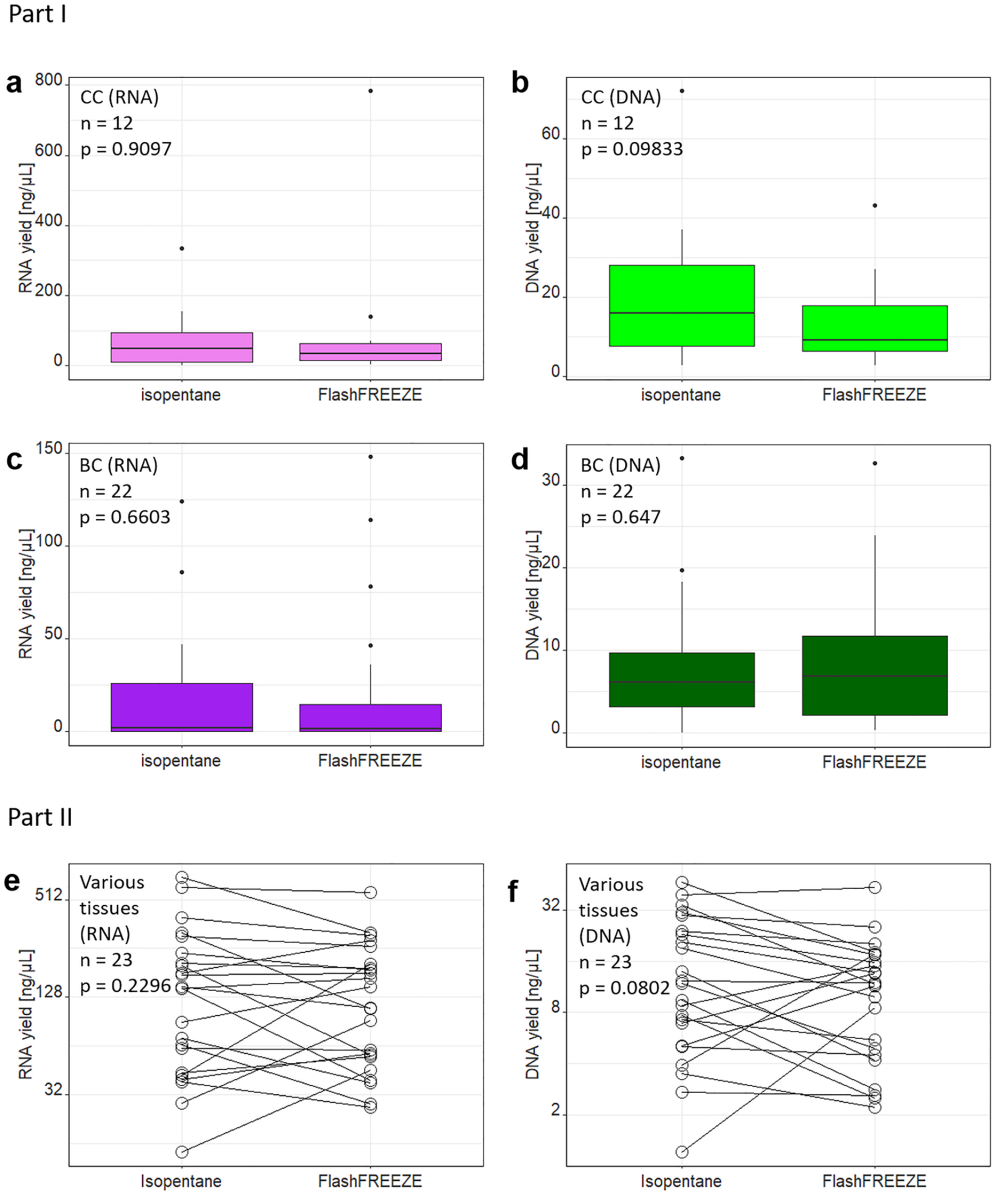
Comparison of the RNA (left column, **a, c, e**) and DNA (right column, **b, d, f**) yield extracted from samples frozen using isopentane or FlashFREEZE unit. Part I: (**a**) and (**b**) colon cancer tissue (CC) (**c**) and (**d**) breast cancer tissue (BC); all isolated using *Protocol_O* (AllPrep DNA/RNA kit; #80204). Part II: (**e**) and (**f**) a set of samples derived from various organs (breast *n* = 12, colon = 6, testis = 2, rectum = 2, uterus = 2), processed using *Protocol_P* (AllPrep DNA/RNA/miRNA kit; #80224; log_2_ scale on* y*-axis for better visibility). Comparison made by Wilcoxon signed rank test or paired* t*-test for RNA and DNA samples respectively

In Part I of the project 24 colon cancer samples (CC; 12 pairs) and 44 breast cancer samples (BC; 22 pairs) were included; RNA and DNA were extracted using *Protocol_O*. No differences were observed in the yield of nucleic acids (whether RNA or DNA) between the two freezing methods (Fig. 1a–d).

Unfortunately, however, RNA amounts obtained from some of the samples (notably from breast cancer tissue) were very low. It indicated that the poor quality might have derived from the extraction method, rather than to the freezing platform, as samples with poor yield were equally represented in both groups. Therefore, we modified the extraction method and samples included in part II of the project, were processed using a different purification kit (AllPrep DNA/RNA Mini kit # 80204 was replaced by AllPrep DNA/RNA/miRNA Universal kit #80224, referred to as *Protocol_P*). Thus, all subsequent experiments were done using samples extracted with *Protocol_P*.

In Part II we included altogether 27 sample pairs from various tissue types (breast cancer, *n* = 12, colon cancer, *n* = 8; testis cancer, *n* = 3; rectum cancer, *n* = 2; corpus uteri, *n* = 1 and adrenal glands *n* = 1; see Table 1).

**Fig. 2:**
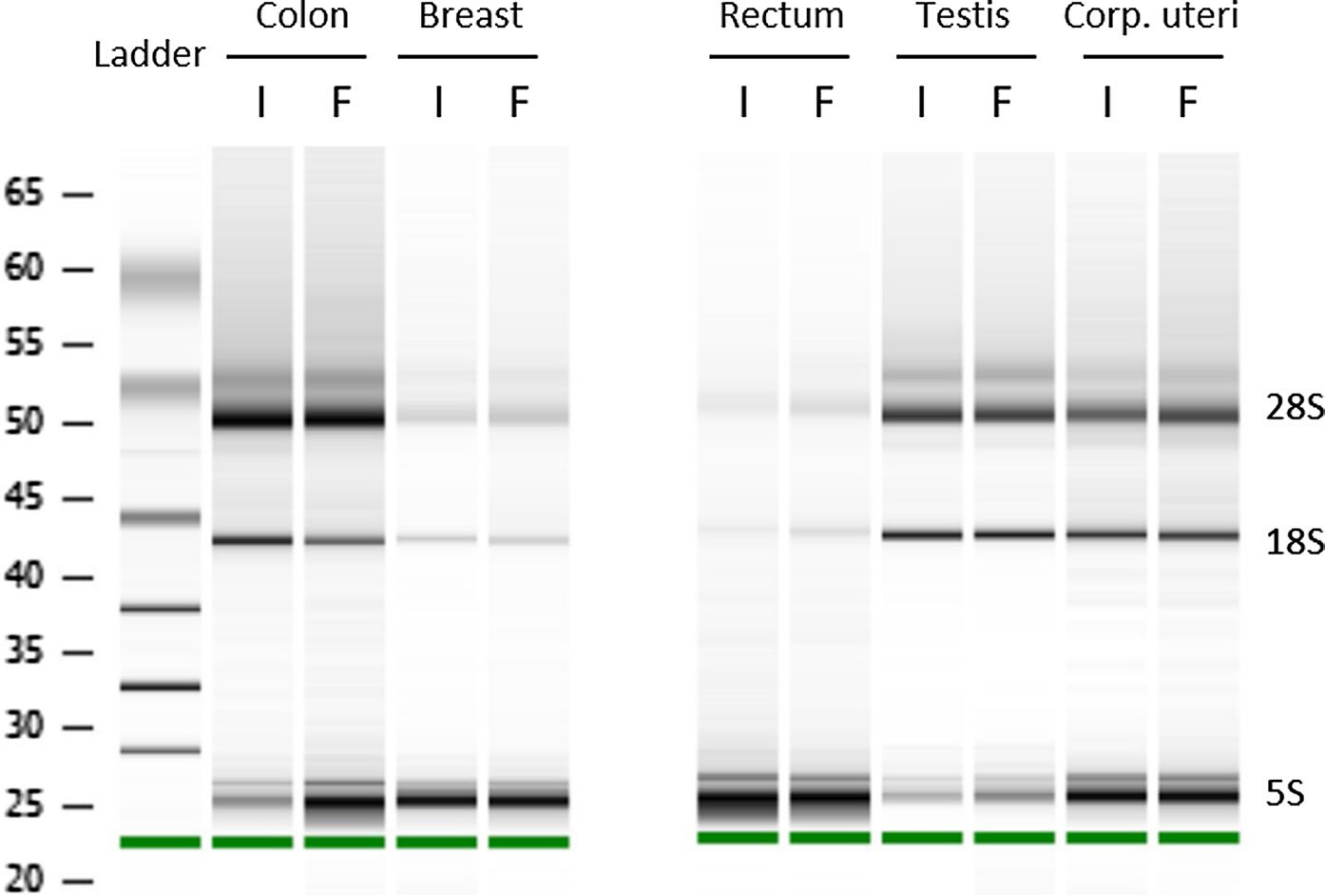
RNA integrity does not differ depending on the freezing method. Results of microcapillary electrophoresis shown as a gel-like image for corresponding isopentane (I) and FlashFREEZE (F) samples

Quantities of total RNA and DNA yield extracted with *Protocol_P* from biospecimens frozen using isopentane or FlashFREEZE unit were in concordance. Differences between RNA/DNA yield within sample pairs seemed to have a random character and did not reach statistical significance (*p* = 0.2296 and *p* = 0.0698 for RNA and DNA, respectively; *n* = 23 sample pairs; four sample pairs were excluded due to a technical error during RNA purification; Fig. 1e and f). Of note, both RNA and DNA yield differed also between sample groups originating from different organs, with breast cancer tissue typically giving lowest yield (Kruskal-Willis test; *p* = 0.0013 and *p* = 0.0034 for RNA and DNA respectively; see also Fig. 2). All these samples showed high DNA and RNA purity, irrespective of the freezing method (Table 2).

**Table 2:** Comparison of DNA and RNA quality from tissue samples frozen with different methods

Parameter of interest	Freezing method	
	Isopentane	FlashFREEZE
260/280 ratio, RNA *n* = 23 pairs	1.91 ± 0.10	1.91 ± 0.10
260/280 ratio, DNA *n* = 23 pairs	1.77 ± 0.11	1.79 ± 0.13
28S/18S ratio *n* = 15 pairs	1.55 ± 0.55	1.54 ± 0.34

Results are expressed as mean ± SD. Comparison made by paired* t*-test

Subsequently, we performed RNA integrity analysis by microcapillary electrophoresis using a Bioanalyzer. Samples isolated with *Protocol_P* showed a distinct peak of low molecular weight RNA (5S peak), which impeded calculating the RNA Integrity Number (RIN). The flat baseline between 5S and ribosomal peaks suggests that the observed peak is due to the presence of small RNAs, rather than RNA degradation (Agilent customer service, personal communication). Importantly, the same pattern has been observed irrespectively of the freezing method used (Fig. 2), and in all tested organs. Therefore, a 28S/18S ratio has been used to evaluate RNA integrity, rather than RIN, and was comparable for isopentane and FlashFREEZE samples (Table 2). RNA integrity analysis was only performed for samples included in Part II (see Table 1). In 8 out of 23 samples isolated with *Protocol_P* peak 5S was so prominent that the lane was otherwise virtually empty, making the analysis inconclusive. However, in the samples where 28S and 18S peaks were visible, they were clearly distinguishable, with no signs of RNA degradation, indicating high quality RNA, suitable for downstream applications (Fig. 2, Table 2).

### Biospecimens yield genetic material equally suitable for molecular testing irrespective of freezing technique applied

To test if the sample freezing method affected results of molecular analyses performed routinely in our diagnostic laboratory, sample pairs were analyzed using NGS sequencing panels (DNA and fusions), qPCR, NGS transcriptome analysis, Affymetrix GeneChip Array and microsatellite instability analysis by fragment analysis.

### NGS sequencing panels, DNA and fusions

**Fig. 3:**
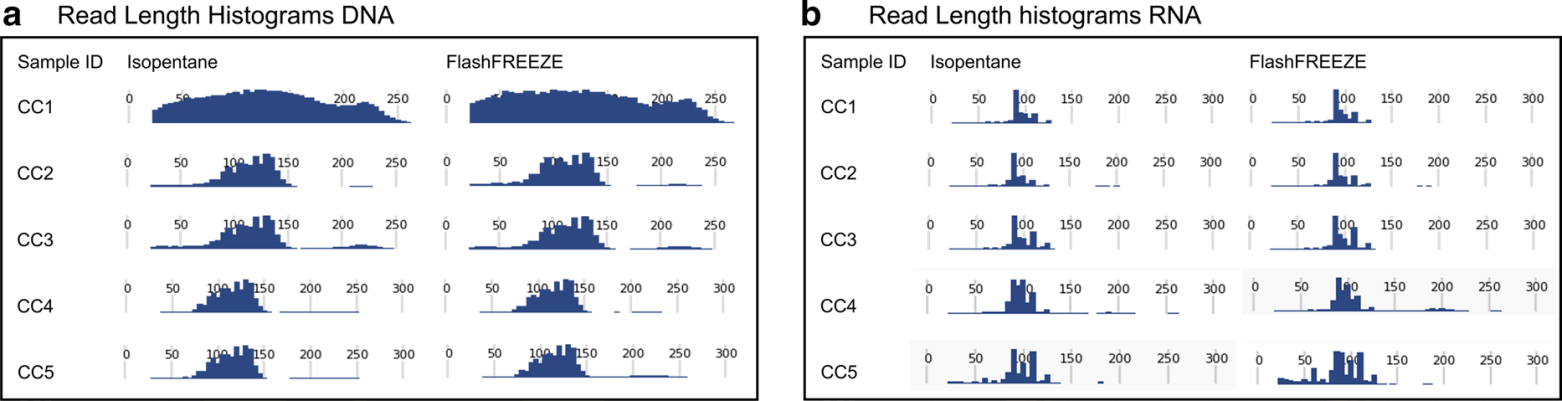
Read Length Histograms from NGS analyses. No difference in Read Length Histograms were found between Isopentane and FlashFREEZE treated samples analyzed with (**a**) Oncomine Focus DNA NGS panel or (**b**) Oncomine Focus RNA NGS panel. The Read Length Histograms of the DNA analysis in sample CC1 showed a failed analysis for both freezing methods

Five tissue sample pairs from colon cancer (CC) (sample ID CC1–CC5) were analyzed using the Ion torrent Oncomine Focus Assay on DNA and RNA (Table 3). Four sample pairs performed equally well on DNA assay with both freezing methods as evaluated on our QC parameters: read length histogram and Median Absolute Pairwise Difference (MAPD), and had the same mutation status within each sample pair. One sample pair (CC1) however repeatedly failed the Focus DNA analysis, indicating a failure during DNA-purification or inhibition in this sample rather than an effect of the sample freezing method (Table 3). All five RNA sample pairs passed all QC parameters of the fusion analysis equally well in material from both freezing methods (Table 3 and Fig. 3). In one RNA sample pair (CC4) a *MET* exon 14 skipping was detected with a low normalized read count level in both samples (Normalization count within gene CC4-I: *MET*(13)–*MET*(15) 213 reads/70925 wild type reads = 0.003, CC4-F: *MET*(13)—*MET*(15) 1883 reads/164940 wild type reads = 0.01). Initially, this event was only detected in one of the samples (CC4-I), but repetition of the analysis with a higher number of total reads confirmed the presence of *MET* exon 14 skipping at a low level in both samples.

**Table 3:** Oncomine Focus NGS data for paired tissue samples, frozen using isopentane (I) or FlashFREEZE unit (F)

Sample ID	DNA			RNA			
	Read length histogram	MAPD	Mutation detected	Read length histogram	Fusion Sample QC	Expression Controls QC	Fusion overall Call
CC1-I	Not acceptable	NA	NA	OK	PASS	PASS	No fusion detected
CC1-F	Not acceptable	NA	NA	OK	PASS	PASS	No fusion detected
CC2-I	OK	0.296	*BRAF* p.Val600Glu	OK	PASS	PASS	No fusion detected
CC2-F	OK	0.363	*BRAF* p.Val600Glu	OK	PASS	PASS	No fusion detected
CC3-I	OK	0.231	None	OK	PASS	PASS	No fusion detected
CC3-F	OK	0.231	None	OK	PASS	PASS	No fusion detected
CC4-I	OK	0.370	*KRAS*, p.Gly12Val	OK	PASS	PASS	*MET*(13)—*MET*(15)
CC4-F	OK	0.288	*KRAS*, p.Gly12Val	OK	PASS	PASS	*MET*(13)—*MET*(15)
CC5-I	OK	0.295	*BRAF* p. Val600Glu, *PIK3CA* p. Arg38His	OK	PASS	PASS	No fusion detected
CC5-F	OK	0.308	*BRAF* p. Val600Glu, *PIK3CA* p. Arg38His	OK	PASS	PASS	No fusion detected

### Suitability for qPCR, with special focus on amplification efficiency and linearity

Ten sample pairs (breast: *n* = 4, colon: *n* = 3, testis: *n* = 1, rectum: *n* = 1, and adrenal glands: *n* = 1) were analyzed with the V600R reaction from the *BRAF* Codon 600 Mutation Analysis Kit II (EntroGen), which is validated for in vitro diagnostics use. As we did not detect *BRAF* V600R mutations in the material used in our study, we focused only on the amplification of the internal control gene. We performed tenfold serial dilutions of each sample, calculated the linearity (*R*^2^ coefficient) and efficiency (E =  −1 + 10^(−1/slope)^) of PCR reaction for each sample, and compared threshold cycle (Ct) values for each pair of samples for the highest amount of starting material (20 ng DNA per reaction).

**Fig. 4:**
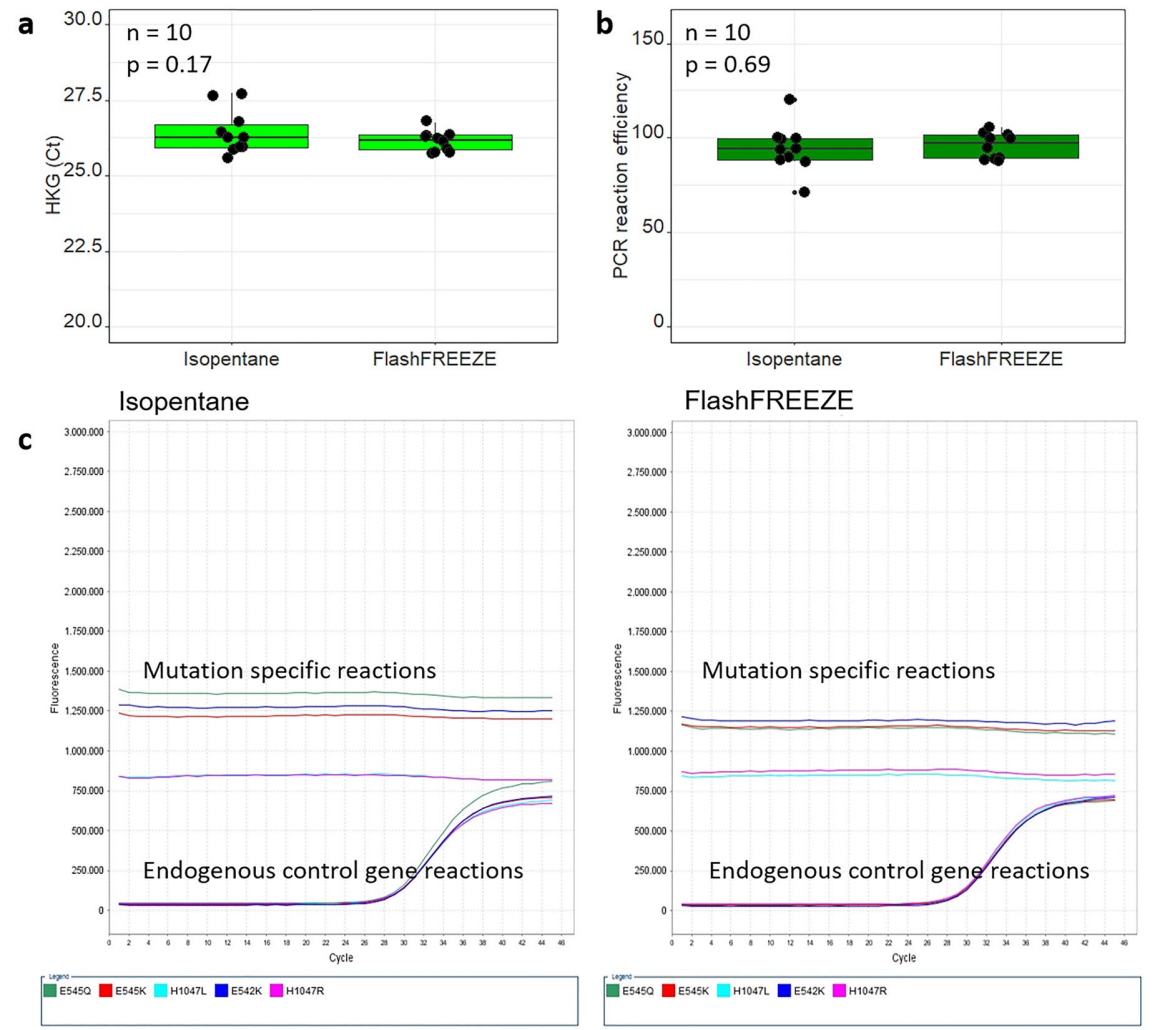
DNA isolated from biospecimens frozen either using isopentane or FlashFREEZE unit can be successfully used for qPCR analysis. (**a**) detection of a house keeping gene (internal control) in DNA samples by qPCR; Ct values for the highest dilution (20 ng DNA/sample) (**b**) efficiency (E) of the qPCR reaction, calculated as E =  −1 + 10^(−1/slope^ where the “slope” refers to the slope of a linear function between log_10_ of DNA concentrations and corresponding Ct values, (**c**). Performance of DNA from a breast cancer sample pair (BC1) in qPCR using *PIK3CA* Mutation analysis kit. Left panel: amplification curves from Isopentane treated sample. Right panel: amplification curves from FlashFREEZE treated sample

Ct values for corresponding Isopentane and FlashFREEZE samples were similar (median Ct difference: 0.15; mean Ct difference: 0.44 between pairs; paired *t*-test *p*-value = 0.17; Fig. 4a) and all samples showed satisfactory linearity (with coefficient of determination R^2^ exceeding 0.98 in all cases). Efficiency of amplification did not differ (paired *t*-test *p*-value = 0.69) between isopentane and FlashFREEZE protocols, and (apart from two exceptions, one BC and one CC, both frozen with isopentane) oscillated between 85 and 100% (Fig. 4b), indicating absence of PCR reaction inhibitors.

Additionally, we analyzed one sample pair from a breast cancer (BC) tissue for *PIK3CA* mutations with the EntroGen PI3K-RT48 (CE IVD) *PIK3CA* Mutation analysis kit. This kit tests for five *PIK3CA* mutations in five different qPCR reactions that each include testing of an endogenous control gene well as. Amplification curves for each sample are showed in Fig. 4c. Both samples were negative for *PIK3CA* mutations and had similar amplification curves on the internal control gene in all reactions (Fig. 4c).

### mRNA expression analyses

**Fig. 5:**
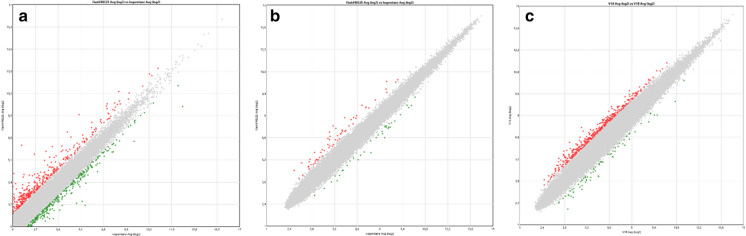
mRNA expression correlation plots (Log_2_ signal) for individual sample pairs. Transcripts with a ± 2 × log_2_ fold changed are marked in green and red (**a**) NGS Transcriptome analysis results from an Isopentane versus a FlashFREEZE preparation of the same breast cancer tumor sample (BC2). (**b**) Affymetrix Gene Chip Array analysis results from an Isopentane versus a FlashFREEZE preparation of the same breast cancer tumor sample (BC3). (**c**) Affymetrix Chip Array analysis results from two different RNA preparations from the same breast tumor FFPE sample, extracted and analyzed in two different analysis runs

mRNA expression was analyzed in two breast cancer tissue sample pairs with NGS transcriptome analysis (BC2; Fig. 5a) and Affymetrix GeneChip Array (BC3; Fig. 5b). Correlation plots of mRNA expression from > 20.000 transcripts (NGS transcriptome analysis) and > 47,000 transcripts (Affymetrix GeneChip Array) are showed in Fig. 5. The mRNA expression found in FlashFREEZE samples was similar to expression levels found in the corresponding samples frozen using isopentane (Fig. 5a and b). For comparative purposes, we have included data from two different RNA preparations from the same tumor FFPE sample, extracted and analyzed in two different analysis Affymetrix GeneChip runs (Fig. 5c). The run to run variation (Fig. 5c) seemed greater than the variation found between different freezing procedures (Fig. 5b), as number of transcripts with a ± log2 fold change was bigger when run-to run variation was evaluated (Fig. 5c, number of transcripts with ± log2 fold change = 467) than when a sample pair subjected to different freezing procedures were analyzed in the same run (Fig. 5b, number of transcripts with ± log2 fold change = 96).

### Microsatellite instability analysis by fragment analysis

**Fig. 6:**
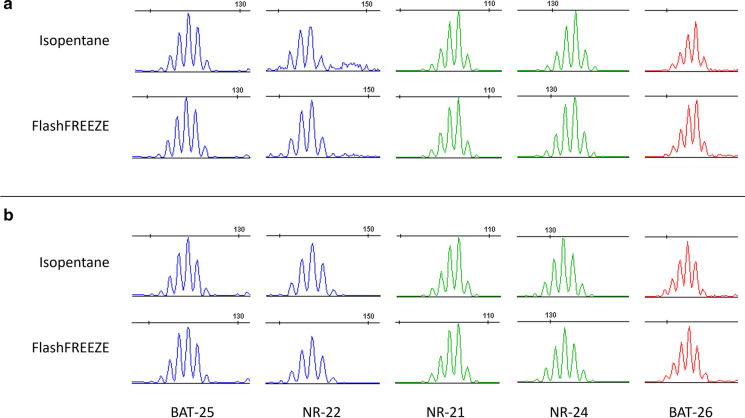
Microsatellite instability analysis. Length of amplicons in each microsatellite loci (BAT-25, NR-22, NR-21, NR-24 and BAT-26) shown for both freezing methods on (**a**) a testis cancer sample and (**b**) a corpus uteri cancer sample. Both samples were microsatellite stable (0 markers exhibiting instability) and the assay worked equally well with DNA obtained from both freezing methods when comparing the length of amplicons obtained with DNA from isopentane treated samples to those observed when applying DNA from the FlashFREEZE treated sample

One sample pair from testis cancer and one sample pair from corpus uteri cancer were randomly selected and analyzed for microsatellite instability with the Pentabase MSI assay (fluochrome coupled fragment analysis). Both samples were microsatellite stable and performed equally independently of the freezing method when evaluated on fragment analysis curves (Fig. 6) and applicability with our standard operational procedure for the analysis (including DNA-input required and dilution of PCR-products prior to fragment analysis etc.)

## Discussion

Medical treatment gradually moves towards precision medicine based on genetic testing, and the availability of molecularly intact biospecimens for diagnostic purposes is essential to avoid bias and obtain credible results. Integrity of nucleic acids (particularly RNA) may be compromised at various steps of tissue handling, and affected by factors such as time to freeze, freezing technique and storing temperature. Stability of RNA and DNA differs greatly, which is influenced by their structure (for instance, the 2’-hydroxyl group in ribose makes RNA more sensitive to hydrolysis) and their role in biological processes. DNA is the hereditary material and carrier of genetic information, and therefore maintaining its integrity is evolutionarily prioritized. RNAs are designed to play a role in gene expression, and their turnover is ongoing during cellular processes (Dunckley and Parker 2001; Berg et al. 2002). Hence, special precautions must be taken when handling samples intended for RNA extraction as it starts degrading rapidly as soon as the tissue is extirpated.

Some researchers suggest that changes in RNA profiles, and hence in gene expression data, may to some extent result from cellular processes such as necrosis or transcriptional response to hypoxia, occurring especially during prolonged time to freeze (Huang et al. 2001; Micke et al. 2006). However, bias in the expression data seems to largely result from RNA degradation, underscoring the importance of correct tissue handling. While purified RNA degrades mostly in a linear and uniform fashion, with expression profiles only slightly affected even upon prolonged heat incubation (Opitz et al. 2010), RNA degradation rates in decaying cells and tissues may be uneven, with relative abundance of specific transcripts markedly changing over time. This is illustrated by a study by Gallego Romero et al., that compares gene expression profiles in PBMCs isolated from four individuals and kept at room temperatures for up to 84 h. Samples collected at later time points, and thus experiencing similar degradation levels, were more correlated than data from the same individual across all timepoints. Altogether, over 60% transcripts showed degradation rates differing from the average rate (Gallego Romero et al. 2014).

The impact of RNA degradation on relative transcript abundance and consequently, on the bias in gene expression estimates, depends greatly on the type of endpoint analysis. Quantitative PCR remains relatively robust (Ohashi et al. 2004; Opitz et al. 2010; Hentze et al. 2019) while more complex settings, such as microarray-based studies are extremely sensitive to RNA quality (Gallego Romero et al. 2014).

In our study design three sample pairs had, due to logistical reasons, a prolonged time-to-freeze, though this time remained identical for compared samples in each pair. To ensure quality and credibility of the results we decided to exclude these samples from the downstream analyses and used them exclusively for yield comparison. Due to the high risk of RNA degradation results of molecular testing for samples with prolonged time-to-freeze should be treated with caution and preferably corrected for RNA quality (as suggested for example by (Jaffe et al. 2017)).

Variation in gene expression patterns may not only result from time-to-process or time-to-freeze extent, but also differ between seemingly optimal handling and storing techniques. Passow et al. performed RNAseq for tissue samples stored in RNAlater, certified as a reliable RNA protective agent, versus samples snap-frozen in liquid nitrogen. Surprisingly, they discovered that RNAlater may be substantially altering the physiology of the samples and impacting gene expression in a non-random manner (Passow et al. 2019). On the other hand, Florell et al. presented somewhat contrary findings, claiming superiority of RNAlater over fresh freezing in maintaining RNA integrity, though they admit that the difference may come from the prolonged time-to-process rather than the preserving technique itself (Florell et al. 2001). Similar findings were presented by Hentze et al*.* who also showed that RNA, at least from gynecological cancers, may be slightly better preserved by immersion in RNAlater than by snap-freezing (Hentze et al. 2019).

Eventually, the freezing protocol may influence biospecimens quality. Rapid freezing (snap-freezing) is preferred over slow freezing because it limits ice crystals formation and hence tissue damage, which leads to histological artifacts known as “Swiss cheese artifacts”. Liquid nitrogen is widely used as the coldest fluid commercially available (Scouten 2010). Rapid stabilization of tissue using liquid nitrogen prevents alteration of genetic profiles and, albeit less appreciated, protein phosphorylation profiles (Shabihkhani et al. 2014). However, as it evaporates quickly, the gas layer forming around the specimen may have an insulating effect. Thus, it prevents rapid freezing of the deeper parts of the tissue, especially in case of larger specimens, and the expansion of ice may result in tissue cracking (Creager et al. 2017). Moreover, due to its low boiling point (− 195.8 °C) it is not feasible to work with and presents a noticeable health hazard.

Other liquids such as isopentane or ethanol, precooled with liquid nitrogen or dry ice, are also widely used, and allow freezing the tissue as vitreous (not expanding) ice. Isopentane has the advantage over ethanol that, when used for freezing the biospecimens by direct immersion, it will not penetrate the tissue (Scouten 2010). In our setting, however, the cryovial, rather than the tissue itself was immersed in isopentane.

Overall, these studies demonstrate that the archiving strategy may have an impact on the downstream analysis.

DNA is reportedly more stable than RNA, as demonstrated by successful cloning from several thousand years old remains (Binladen et al. 2006). However, DNA samples should also be handled with care, as it undergoes degradation in unfavorable conditions (high temperature, pH, ionic strength) (Lindahl 1993) or during cell death program development (“apoptotic ladder”).

In this project, we set to ensure that samples frozen using the FlashFREEZE benchtop device would perform similarly in molecular analyses to samples frozen using dry ice-cooled isopentane. To our knowledge there are no publications, reporting the use of FlashFREEZE system in tissue biobanking. From our point of view, the possibility of implementing FlashFREEZE would present a feasible and easy-to-standardize procedure, posing a lesser health hazard and with lesser impact on the environment than isopentane. With a lower coolant consumption and no need of dry ice addition, FlashFREEZE system can also be less costly in the long run, though purchase of the equipment itself is an expense that must be considered.

The number of comparisons was in some assays not sufficient to perform statistical analyses. However, our aim was to include a possibly broad spectrum of analyses, routinely done in our department. We believe that, though in some individual cases the number of samples was low, collectively these data support that the nucleic acids extracted from tissue samples frozen either with isopentane or with FlashFREEZE have similar quality and perform similarly in the endpoint analyses.

## Data Availability

The datasets (raw data) generated during and/or analysed during the current study are available from the corresponding author on reasonable request.
